# Editorial Notes

**Published:** 1893-07

**Authors:** 


### Editorial Notes.

A LABORATORY is to be erected at Calcutta for the purpose of studying the various snake poisons and their antidotes.

THE American Pædiatric Society held its fifth annual meeting at West Point, N. Y., on May 24th, 25th and 26th.

THE American Gynæcological Society held its eighteenth meeting in Pniladelphia, on May 16th, 17th and 18th.

THE wife of Koch, the bacteriologist, has secured a divorce, with alimony to the extent of one-fourth his income. He is to marry a young actress now playing at Barany's theater.

LADY LISTER, the wife of Sir Joseph Lister, died of Pneumonia recently, while traveling in Italy. Lady Lister was the daughter of the late distinguished Edinburg surgeon, Mr. Syme.

THE President's chair of the Massachusetts Surgical and Gynæcological Society has been filled by a woman, Dr. Sarah E. Sherman, of Salem. All the other officers of the Society are men.

THE Grand River Medical Society, at Chillicothe, Mo., on June 1st and 2d, elected the following officers for the ensuing year: President, Dr. H. H. Wilson, Trenton; First Vice-President, Dr. C. F. Brown, Newton; Second Vice-President, Dr. N. W. Wingo, Spring Hill; Secretary, Dr. C. W. Patton, Spring Hill; Assistant Secretary, Dr. C. R. Martin, Hickory; Treasurer, Dr. R. Barney, Sr., Chillicothe. After the selection of Trenton for the next place of meeting the session adjourned.

THE LATE DR. R. O. ENGRAM.—The death, at Montezuma, of Dr. R. O. Engram, editor of *The Record*, is a loss to Georgia journalism and to the State generally. As a physician, no man stood higher in the profession; as an editor, his fearless pen championed the right and did brave work for the State he loved; as a citizen, his character was above reproach. We repeat that the State sustains a loss in his death, and he will not be forgotten. He was "a man among men."

---

THE Arkansas Medical Association held its annual session at Batesville on June 2d, 1893. The following officers were elected for the ensuing year: President, Dr. D. C. Ewing, of Batesville; Vice-Presidents, Adam Guthrie, Jr., Quitman; W. W. Bailey, Fort Smith; E. A. Baxter, Melbourne, and D. L. Jones, Haynes; Secretary, L. P. Gibson, Little Rock; Treasurer, A. L. Breysacher, Little Rock; Librarian, R. B. Christian, Little Rock.

---

THE North Carolina Medical Society met in Raleigh, N. C., on May 9th, 10th and 11th.

The following officers were elected for the ensuing year:

President, Dr. W. H. H. Cobb, of Goldsboro; Vice-Presidents, Dr. J. A. Hodges, of Wilmington, Dr. R. W. Tate, of Greensboro, Dr. Willis Alston, of Littleton, Dr. M. H. Fletcher, of Asheville; Secretary, Dr. R. D. Jewett, of Wilmington; Treasurer, Dr. M. P. Perry, of Macon.

The committee chose as orator for next year, Dr. E. G. Moore, of Toisnot.

The next session will be held at Greensboro, May 15th, 1894.

